# Increased Sociability in Mice Lacking Intergenic *Dlx* Enhancers

**DOI:** 10.3389/fnins.2021.718948

**Published:** 2021-10-04

**Authors:** Siavash Fazel Darbandi, Crystal Esau, Cindy Lesage-Pelletier, Simon Monis, Luc Poitras, Man Yu, Sofia Perin, Gary Hatch, Marc Ekker

**Affiliations:** Department of Biology, University of Ottawa, Ottawa, ON, Canada

**Keywords:** autism, development, *Dlx*, GABA, gene regulation, Williams–Beuren syndrome

## Abstract

The *Dlx* homeodomain transcription factors play important roles in the differentiation and migration of GABAergic interneuron precursors. The mouse and human genomes each have six *Dlx* genes organized into three convergently transcribed bigene clusters (*Dlx1/2*, *Dlx3/4*, and *Dlx5/6*) with *cis*-regulatory elements (CREs) located in the intergenic region of each cluster. Amongst these, the I56i and I12b enhancers from the *Dlx1/2* and *Dlx5/6* locus, respectively, are active in the developing forebrain. I56i is also a binding site for GTF2I, a transcription factor whose function is associated with increased sociability and Williams–Beuren syndrome. In determining the regulatory roles of these CREs on forebrain development, we have generated mutant mouse-lines where *Dlx* forebrain intergenic enhancers have been deleted (I56i^(–/–)^, I12b^(–/–)^). Loss of *Dlx* intergenic enhancers impairs expression of *Dlx* genes as well as some of their downstream targets or associated genes including *Gad2* and *Evf2*. The loss of the I56i enhancer resulted in a transient decrease in GABA^+^ cells in the developing forebrain. The intergenic enhancer mutants also demonstrate increased sociability and learning deficits in a fear conditioning test. Characterizing mice with mutated *Dlx* intergenic enhancers will help us to further enhance our understanding of the role of these *Dlx* genes in forebrain development.

## Introduction

*Dlx* genes encode homeodomain transcriptional regulators that play multiple roles during embryonic development, notably in the forebrain, developing craniofacial skeleton and teeth, sensory organs and limbs. Mice have six *Dlx* genes, *Dlx1–6*, four of which *Dlx1*, *Dlx2*, *Dlx5*, and *Dlx6* are expressed in the developing forebrain. *Dlx* transcripts are mainly found in the ventral telencephalon and diencephalon of all vertebrates examined thus far with highly overlapping patterns (Simeone et al., 1994; Ellies et al., 1997; Liu et al., 1997). Thus, targeted mutations causing loss of function of individual *Dlx* genes often result in subtle phenotypes. However, when multiple *Dlx* genes are mutated such as in the case of the *Dlx1^(–/–)^ – Dlx2^(–/–)^* double mutants, a more severe phenotype is observed that includes impaired migration and differentiation of GABAergic interneuron precursors (Anderson et al., 1997a,b).

The six mouse *Dlx* genes are organized as three convergent bigene clusters *Dlx1/Dlx2*, *Dlx3/Dlx4*, and *Dlx5/Dlx6* with relatively short intergenic regions (Stock et al., 1996; Ellies et al., 1997). The *Dlx* bigene clusters are arranged in a convergent configuration with some of the enhancers located within the intergenic region. We have previously identified highly conserved enhancer elements in the *Dlx1/Dlx2* and in the *Dlx5/Dlx6* intergenic regions (Zerucha et al., 2000; Ghanem et al., 2003). For example, the I56i and I56ii enhancers from the *Dlx5/Dlx6* locus faithfully target expression of reporter transgenes to the forebrain (Zerucha et al., 2000). Similarly, the I12b sequence with forebrain enhancer activity was found in the *Dlx1/Dlx2* intergenic region (Ghanem et al., 2003). The extent of sequence conservation between species is remarkable (more than 90%) for the I56i enhancer and the mouse and human sequences are nearly identical over 450 bp (Zerucha et al., 2000; Ghanem et al., 2003). The forebrain enhancer activity can be observed in both transgenic mice and zebrafish and is observed with either the mouse, human, or zebrafish sequences, confirming that sequence conservation also results in functional conservation (Zerucha et al., 2000; Ghanem et al., 2003). Sharing of *cis*-regulatory elements (CREs) between the two members of a *Dlx* bigene cluster may contribute to the overlap in gene expression and to their partial functional redundancy. Furthermore, several lines of evidence suggest that *Dlx* genes are involved in auto- and cross-regulatory interactions and that intergenic CREs play a role in these processes (Zerucha et al., 2000; Zhou et al., 2004; Poitras et al., 2007, 2010; Yu et al., 2021). Interestingly, one of the main factors binding to the I56i enhancer is GTF2I (Poitras et al., 2010), a factor associated with hypersociability in dogs (vonHoldt et al., 2017) and part of the Williams–Beuren syndrome locus (Francke, 1999).

Here, we examine the consequences of deleting the I56i enhancer and/or the I12b enhancer. We also replaced the I56i enhancer with a variant that includes a SNP (adenine to guanine substitution in a highly conserved region of I56i, position 182) that was identified in a family with cases of autism and that affects enhancer activity in reporter constructs (Hamilton et al., 2005; Poitras et al., 2010). Mutating or deleting I56i impairs *Dlx* expression as well as that of downstream targets such as *Gad* genes that encode the enzyme glutamic acid decarboxylase responsible for GABA synthesis. Changes in gene expression were accompanied by decreases in the number of GABAergic neurons and behavioral abnormalities that were observed in adults. Deletion of the I56i enhancer had a bigger impact than that of I12b; however, the combinatorial deletion of enhancers from both the *Dlx1/Dlx2* and *Dlx5/Dlx6* bigenes (I12b-I56i and I12b-vI56i) resulted in more profound phenotypes.

## Materials and Methods

### Animals

Mice were group-housed in standard mouse cages in a room with a 12 h light–dark cycle and *ad libitum* access to food and water and all animal experiments were approved by the Standard Operating Procedures and Guidelines of the uOttawa Animal Care Committee. The mice were anesthetized with CO_2_, followed by a vertebral dislocation. All researchers interacting with these mice obtained the required National Institute for Animal User Care Training (NIAUT).

### Generation of Mice With Targeted Enhancer Deletion or Mutation

**FIGURE 1:**
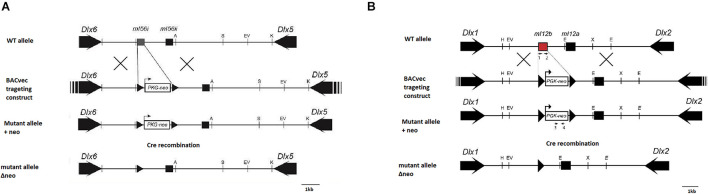
Targeting strategy for the generation of mutant mice with deletions of forebrain *Dlx* enhancers. **(A)** Strategy for the deletion of enhancer I56i. **(B)** Strategy for the deletion of enhancer I12b. Homologous recombination in embryonic stem cells was completed using a BAC targeting construct wherein the enhancer was replaced with a floxed neomycin cassette. Recombinant clones were transferred into a blastocyst to generate chimeric embryos. Mice positive for the recombinant *Dlx* loci were crossed with CRE mice to remove the neomycin cassette. Black arrows, transcriptional orientation of each gene; black triangles, lox p locations. A, *Age*I; H, *Hin*dIII; S, *Sal*I; E, *Eco*RI; EV, *Eco*RV; K, *Kpn*I; X, *Xba*I.

The strategy and target vector used to produce mice lacking the I56i enhancer (I56i^(–/–)^) is depicted in Figure 1. Through homologous recombination, a LoxP-flanked PGK-neomycin-resistant cassette replaced the entire I56i enhancer on a Bacterial Artificial Chromosome (BAC) harboring the *Dlx5/Dlx6* locus. The BAC was screened and sequenced to ensure recombination occurred.

For the mice having the SNP in I56i corresponding to that found in a family with case of autism (Hamilton et al., 2005), vI56, a modified enhancer-neomycin resistance cassette was inserted in place of the wild-type I56i enhancer present on a BAC containing the *Dlx5/Dlx6* locus. This was accomplished by homologous recombination in bacteria as described in Lee et al. (2001). Clones positive for neomycin were selected with G418, and 100 embryonic stem (ES) cell colonies were screened for the correct gene using quantitative real-time PCR (Feng et al., 2006). The A to G mutation was verified by sequencing the genomic DNA of positive ES clones. One clone was correctly targeted and used to generate the “knock-in” mice.

For mice lacking the I12 enhancer (I12b^(–/–)^, Figure 1), a BAC clone (#510G1) containing a ∼200 kb *Dlx1/Dlx2* locus was obtained by screening a BAC library (BACPAC Resources Center) made from the liver tissue of strain 129/Sv mice. To generate the I12b targeting vector (I12b-510G1), a modified LoxP-flanked neomycin (neo) selection cassette driven by a murine PGK promoter was inserted into the place of the wild-type I12b enhancer present on the isolated BAC clone, which was accomplished by homologous recombination in *E. coli* EL250 at 32°C as described in Lee et al. (2001). The recombined BAC clones were then verified for sequence accuracy at the site of recombination prior to the electroporation into ES cells.

Mutant mice were generated at the Transgenic Mouse Core Facility at the McGill Cancer Center. The engineered BACs were electroporated into 129Sv mouse ES cells and positive clones were selected with gentamicin. ES cells were screened for the presence of the neomycin cassette through quantitative real-time PCR. A positive ES clone was injected into a host C57BL/6N blastocyst to generate chimeric mice. Chimeras were selectively mated with C57BL/6N wild-type mice and genotyped for the production of heterozygote progeny that were positive for the targeted mutations in their germ line. Backcross with C57BL/6N wild-type mice was done for a minimum of 3 generations.

### Genomic DNA Extraction (Genotyping)

Tissue samples were digested in 200 μL of digestion cocktail, containing 10 μL of 10 mg/mL proteinase K per 100 μL of digestion buffer (50 mM Tris–HCl, pH = 8.0, 100 mM EDTA, 100 mM NaCl, and 1% SDS). Genomic DNA was extracted using a standard salt and ethanol precipitation protocol. A PCR-based approach was used to screen for mutant embryos by using 1 μL of purified genomic DNA, and primer-pairs flanking each deleted enhancer (Table 1).

**TABLE 1:** Primers used for genotyping.

ΔI12b: FWD – tgagtctgtaatggcaaaatgc; REV – caggtgcagattcccgaag
ΔI12b: FWD – ggaaaatgcaattttggga; REV – caggtgcagattcccgaag
ΔI56i: FWD – cattgggagcccagttctaa; REV – caatatccccgttccctttt
ΔI56i: FWD – cagttctaagcagagttctag; REV – ctcagtcagtctgaatgg
ΔI56ii FWD – gagggaagaaagacgggagt; REV – gtcagagcccaaaccttgaa
ΔI56ii FWD – acggaagcaagacaggcaag; REV – gaggtggctttggtggagag
SNP65i Scr2: FWD gcttcaaattggatggcact; REV – tacagacctgggcatccttc
SNP56i Scr3: FWD – ccccaatgtctgcttcaaat; REV – ggaagccccatactgtgaga

### RNA Extraction and cDNA Synthesis

Total RNA was extracted from the ventral telencephalon of wild-type and homozygote embryo at various embryonic stages using QIAGEN RNeasy Plus^®^ Kit following manufacturer’s protocol. First strand cDNA was synthesized using superscript reverse transcriptase II following manufacturer’s protocol (Invitrogen Life Technologies).

### Quantitative Real-Time PCR

Quantitative real-time PCR (qRT-PCR) was performed using SYBR Green (Bio-Rad) and an Illumina real time cycler (MBI Lab Equipment). Gene-specific primers for *Dlx* genes (*Dlx1*, *Dlx2*, *Dlx5*, and *Dlx6*), Gad1, *Gad2*, long non-coding RNA (lncRNA) *Evf2*, and *ef1*α housekeeping gene were designed using the Primer 3 program (Rozen and Skaletsky, 2000; Table 2). The expression level of the target genes were measured relative to the expression levels of *ef1*α and wild-type littermate as previously described (Darbandi and Franck, 2009).

**TABLE 2:** Primers used for qRT-PCR experiments.

Primer name	Primer sequence (5′–3′)
*Ef1a.For*	AAGCTCTTCCTGGGGACAAT
*Ef1a.Rev*	ATGCTATGTGGGCTGTGTGA
*Dlx1.For*	CAGTTGCAGGCTTTGAACC
*Dlx1.Rev*	ACTTGGAGCGTTTGTTCTGG
*Dlx2.For*	GCCTCACCCAAACTCAGG
*Dlx2.Rev*	GCCGCTTTTCCACATCTTC
*Dlx5.For*	CGACTTCCAAGCTCCGTTC
*Dlx5.Rev*	TTCTTTCTCTGGCTGGCTG
*Dlx6.For*	CGGACCATTTATTCCAGCC
*Dlx6.Rev*	CGCTTATTCTGAAACCATATC
*Gad2.For*	TCATTGCCCGCTATAAGATG
*Gad2.Rev*	GCAGCTCCCTTCTTGAGAGA1

#### Histology

Embryos from the mating of a deletion C57BL/6N heterozygote male with heterozygote female were harvested at various embryonic stages. E13.5 and E14.5 mouse embryos were fixed in 4% paraformaldehyde (PFA) in 1× PBS overnight at 4°C. The brains were transferred into 30% sucrose and incubated overnight at 4°C. Following the sucrose treatment, the brains were washed in 1× PBS for 5 min at RT and embedded in Tissue Tek^®^ O.C.T. compound. Twenty micrometers sections were obtained from the embedded specimen, utilizing a LEICA CM1850 cryostat-microtome and collected using Superfrost^®^ Plus microscope slides (Fisherbrand).

### Immunohistochemistry

Frozen sections of E13.5 and of P35 mouse brain were obtained as described above and air dried for 2 h at RT, then incubated in 350 μL 1× PBST to eliminate residues from tissue protection medium. Immunostaining was performed as described earlier (Ghanem et al., 2007). The following antibodies have been applied in this study: rabbit anti-GABA (1:500, Sigma), rabbit polyclonal anti-calbindin (1:1000), mouse polyclonal anti-parvalbumin (1:1000). For fluorescent imaging, histological sections were cover-slipped with a Vectashield mounting medium (Vector Labs), analyzed with a Zeiss Axiophot fluorescence microscope. The somatosensory cortex of P35 mice was imaged. Four to five sections were examined for each animal with a minimum of three animals per genotype.

#### *In situ* Hybridization

*In situ* hybridization on frozen tissue sections and digoxigenin RNA probe labeling were performed according to the procedures described in Fazel Darbandi et al. (2016) and Smith et al. (2008). Hybridized probes were detected with an AP-conjugated anti-digoxigenin Fab fragment antibody (1:2000, Roche) and visualized with the NBT/BCIP substrate system. Antisense riboprobes for *Dlx* genes (*Dlx1*, *Dlx2*, *Dlx5*, and *Dlx6*), TF’s including *Islet1*, *Meis2*, *Gad2*, and ncRNA *Evf2* were prepared as previously described (Fazel Darbandi et al., 2016).

### Behavioral Analysis of Mutant Mice

The following tests were carried out in the following order: beam break, elevated plus maze, open field, juvenile interactions and fear conditioning. Between 10 and 20 animals of each genotype were tested between 8 and 13 weeks of age and were compared to wild-type littermates of the same age. Each genotype comprised an equal number of males and females. Testing was performed blind to genotype.

The beam break test measures general motor activity of mice. Mice are placed in a housing cage that is stationed on a metal frame connected to infrared receptors and emitters with a Micromax analyzer (Accuscan). The mice are free to roam the cage for 2 h and their motor activity is tracked by invisible infrared light beams. Photocell emitters located on each side of the cages send horizontal beams of infrared light to the opposite side of the cage and are detected by photocell receptors. As the animal moves through the cage and breaks the beams, the photocell analyzer records the average number of beam breaks over a 2 h period.

The elevated plus maze measures fear and anxiety based on the preference of the animal to explore dark enclosed spaces compared to bright exposed places. The mice are placed one at a time in the center of an elevated four arm maze measuring 5 cm wide and 60 cm long. Two of the arms are open platforms while the other two have enclosed walls measuring 14.5 cm high. The maze is elevated 1 m above ground and the test is performed with the lights on to increase the anxiety of being in an open arm. Once the mice have been placed in the center of the maze they are free to explore for 10 min while the investigator leaves the room. The movement of the mouse is tracked by a camera located above the maze using software from Noldus (Ethovision). The output data is a representation of the number of times the animal has entered into the open and closed arms.

The open field test measures fear, anxiety, and motor function based upon an animal’s desire to explore a novel environment and its fear of exploring a brightly lit open area. The mouse is placed in the upper right corner of each open field box. The square box is 45 cm wide and 45 cm high. After the mouse is placed in the box, it has 10 min to freely explore all areas of the box. The behavior of the mouse is recorded by a video camera located above the box connected to the ceiling. The data output is comprised of the amount of time the mouse has spent in the center of the box in comparison to the corners. The video tracking software used for open field testing is Noldus (Ethovision).

The juvenile interaction test involved two mice, a test animal and a juvenile (21–28 days of age) of the same sex. Test mice were allowed to habituate in the testing room for 15 min prior the test. Then, the test mouse and the juvenile were placed in a novel cage together at the same time, and allowed to explore for 2 min. Total duration of social interactions is scored as cumulative seconds spent by the test mouse in sniffing the nose, anogenital, and other body regions of the juvenile. Three days later, the same two mice were placed together again in a new cages for another 2 min. The time the test mouse spends interacting with the juvenile was again recorded. The interaction time from first exposure to the juvenile and the second exposure to same juvenile are compared. All the trials were performed in darkness under red light with a background noise. Silent stop watches were used to record the 2 min intervals and the cumulative interaction time.

For fear conditioning, the mouse’s freezing reaction is recorded when put into a sudden fearful situation. This test takes 3 days. The first day is dedicated to “training” the mice. In brief, the mouse is placed in the fear conditioning cage for 6 min in which they are free to explore. After 2 min, a tone is played from within the apparatus for 30 s, ending with a 2 s 0.5 milliamp shock. One minute after the shock the tone is repeated for 30 s followed by another 0.5 milliamp shock for 2 s. In the last 2 min, there is no tone or shock. Over the duration of the 6 min, the freezing behavior of the mouse is recorded. The second day of testing is contextual conditioned fear testing and must be done 24 h after day 1. The mouse is placed in the same apparatus with identical lighting and room conditions as day 1. The mouse’s freezing behavior is recorded for 6 min and measures the fear associated with being placed back into the same environment where an adverse stimulus (shock) was delivered. On the third day of testing, the mouse is placed in a novel environment with a novel smell and light. Once the mouse is placed in the novel environment, its freezing is recorded for 3 min to ensure the animals do not associate the novel environment from the environment on days 1 and 2. After 3 min, the tone that was played on day 1 is played for 30 s and the freezing is recorded, measuring the fear associated with the tone.

Detailed protocols for all behavioral tests can be obtained from the authors upon request.

### Statistical Analysis

All statistical analysis was performed using the software GraphPad Prism v7.0 (San Diego, CA, United States). For gene expression analysis, significance was quantified using multiple *t*-test and Holm–Sidak analysis. For the juvenile interaction and fear conditioning tests, one-way ANOVA with Turkey’s multiple comparison test was done. Statistical significance was determined using a 95% confidence interval where *p* < 0.05. ^∗^*p* < 0.05, ^∗∗^*p* < 0.01, ^∗∗∗^*p* < 0.001.

## Results

### Mice With *Dlx* Enhancer Deletion Do Not Exhibit Any Morphological Abnormalities

We generated mice with targeted deletion of the I56i intergenic enhancer (*Dlx5/6* locus), of the I12b intergenic enhancer (*Dlx1/2* locus) both of which showing forebrain enhancer activity that recapitulates *Dlx* expression (Zerucha et al., 2000; Ghanem et al., 2003). We also produced mice where the I56i with a SNP variant that has been associated with cases of autism (Hamilton et al., 2005) and that affects enhancer activity in transgenic mice (Poitras et al., 2010).

Mice that are homozygous for a single targeted deletion of I56i, I12b, or that have the variant I56i enhancer (vI56i) are viable, fertile, and do not show obvious developmental defects. Thus, there were no apparent changes in embryonic and neonate body size (examined at E11.5, E14.5, and P0, more than 30 mice per time point) nor in brain size when examined at P0. No differences between sexes were observed. Similarly, mice that carry a combinatorial deletion of I12b and either I56i deletion or the vI56i are also viable.

### Altered Gene Expression in Mice Lacking the I56i Intergenic Enhancer

The I56i enhancer from the *Dlx5–Dlx6* bigene cluster has activity in the subventricular zone (SVZ) and mantle zone (MZ) of the developing telencephalon starting at E11.5 (Zerucha et al., 2000; Ghanem et al., 2003, 2007). We used RT-qPCR and *in situ* hybridization to investigate the impact of I56i deletion on the expression of the *Dlx* genes and other associated genes, namely *Gad1/2* and *Evf2*.

**FIGURE 2:**
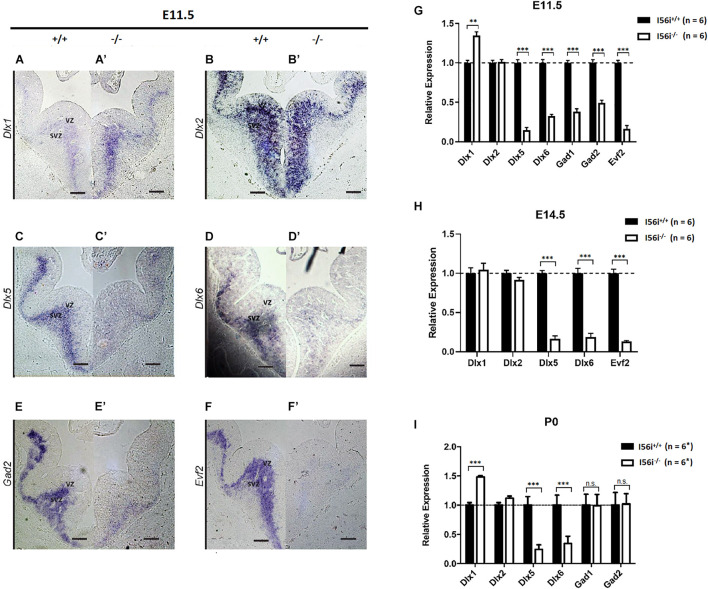
*Dlx* and *Dlx* target gene expression are reduced in the developing forebrain of ΔI56i mice. **(A–F,A′–F′)** *In situ* hybridization on the ventral telencephalon at E11.5 for **(A,A′)** *Dlx1*, **(B,B′)** *Dlx2*, **(C,C′)** *Dlx5*, **(D,D′)** *Dlx6*, **(E,E′)** *Gad2*, and **(F,F′)** *Evf2*. **(A–F)** WT mice and **(A′–F′)** homozygous ΔI56i mice. Scale bar = 50 μm. **(G–I)** qRT-PCR of ventral telencephalon isolated from WT and homozygous I56i^(–/–)^ mice. **(G)** E11.5, **(H)** E14.5, and **(I)** P0. Bars are WT (black), homozygous I56i^(–/–)^ (white). Data presented as mean for the *n* values as indicated except for *Gad1* and *Gad2* at P0 for which *n* = 3. Error bars represent SEM. Data analyzed using a two tailed *t*-test (**p < 0.01, ***p < 0.001). SVZ, subventricular zone; VZ, ventricular zone.

Mice harboring I56i^(–/–)^, show ∼40% increase in *Dlx1* expression in the telencephalon at E11.5 and P0 (Figures 2A,G,I). This increase may be related to compensatory mechanisms in response to the large decreases in *Dlx5/Dlx6* expression seen in those mutants at. E11.5, E14.5, and P0 (Figures 2C,D,H,I). Expression of *Dlx2* was unaffected (Figures 2B,G and data not shown).

We next looked at the expression of genes that are targets of *Dlx5/6* such as *Gad1* and *Gad2* that code for the glutamic acid decarboxylases necessary for GABA production. Expression of these two genes was markedly decreased during embryonic development (E11.5 and E14.5; Figures 2E,G,H). However, expression of both *Gad1* and *Gad2* had returned to WT levels when measured at P0 (Figure 2I or data not shown).

We examined impact of I56i enhancer deletion on the expression of the lncRNA *Evf2* (Feng et al., 2006), which is located at the *Dlx5/6* locus and this was found to be near undetectable levels (Figures 2F–H), presumably because the I56i deletion also removes the promoter of this gene (Figure 1).

The I56i enhancer is also the site of a SNP (adenine to guanine substitution at position 182 of I56i) that has been found in a family with cases of Autism (Hamilton et al., 2005). This SNP has a deleterious effect on I56i enhancer activity as tested in reporter constructs (Poitras et al., 2010). We took advantage of the remarkable conservation of I56i sequence between mouse and human (Zerucha et al., 2000) to create mice that have the SNP version of the I56i enhancer (vI56i mice).

**FIGURE 3:**
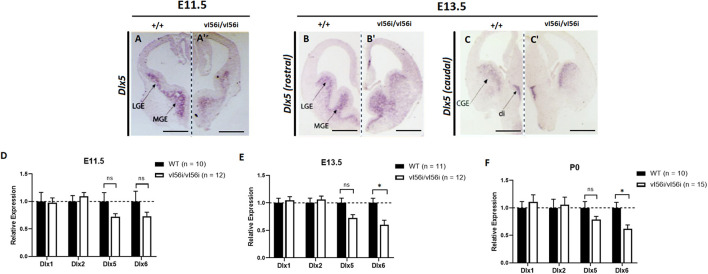
*Dlx* expression is reduced in the developing forebrain of mice homozygous for a variant I56i enhancer. **(A–C,A′–C′)** *In situ* hybridization on forebrain for *Dlx5* at **(A,A′)** E11.5, **(B,B′)** E13.5 rostral slice, and **(C,C′)** E13.5 caudal slice. **(A–C)** WT mice and **(A′–C′)** homozygous vI56i mice. Scale bar = 1 mm **(A,A′)** and 1 mm **(B,C,B′,C′)**. LGE, lateral ganglionic eminence; MGE, median ganglionic eminence; CGE, caudal ganglionic eminence; Di, diencephalon. **(D–F)** qRT-PCR of ventral telencephalon isolated from WT and homozygous vI56i mice. **(D)** E11.5, **(E)** E13.5, and **(F)** P0. WT (black), homozygous vI56i (white). Data presented as mean for the *n* values listed in brackets. Error bars represent SEM. Data analyzed using a two tailed *t*-test (*p < 0.05).

We examined the impact of vI56i on the expression of *Dlx5* and of *Dlx6* at E11.5, E13.5, and P0, We observed decreases that ranged from 20 to 40% but these reached statistical significance only for Dlx6 at E13.5 and P0 (Figures 3D–F) reached statistical significance (Figure 3). Expression of *Dlx1* and *Dlx2* was unaffected.

The I12b enhancer from the *Dlx1/Dlx2* bigene cluster shows remarkable similarities in its activity patters in the telencephalon when compared to that of I56i when tested in reporter constructs despite the two enhancers showing no overall similarities in overall DNA sequence (Ghanem et al., 2007). We generated mice lacking the I12b sequence, ΔI2b. We also wondered if deletions of the I12b enhancer might further exacerbate the effects of deleting I56i on *Dlx* expression.

**FIGURE 4:**
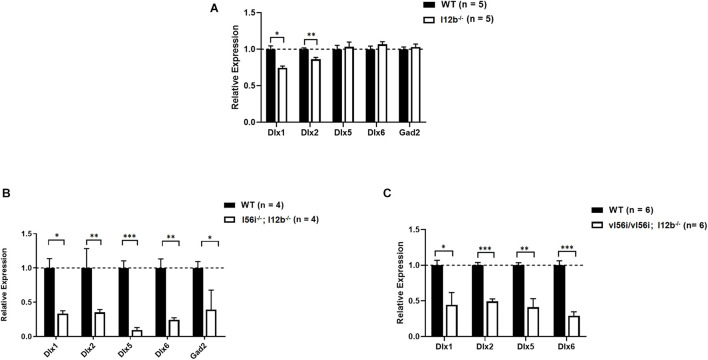
I12b enhancer deletion and combined I12b/I56i mutations impairs *Dlx* and *Gad2* expression levels. qRT-PCR of ventral telencephalon isolated from WT mice and mice **(A)** I12b^(–/–)^, **(B)** homozygous for both the I12b and I56i deletions, and **(C)** homozygous for both I12b^(–/–)^ and vI56i. Bars are WT (black), mutant (white). Data presented as a mean for the *n* values listed in brackets. Error bars represent SEM. Data analyzed using a two tailed *t*-test (*p < 0.05, **p < 0.01, ***p < 0.001).

Mice homozygous for the I12b deletion showed decreases in *Dlx1* and *Dlx2* expression at E13.5 (Figure 4A). Thus, similar to the I56i deletion, the I12b deletion impacted genes at the same locus but the impact was much milder that of I56i deletion on the associated *Dlx5* and *Dlx6* genes (Figure 2). Deletion of I12b did not affect expression of *Dlx5*, *Dlx6*, and other targets including *Gad2, Mash1*, *Nkx2*.1, or *Gsh2* (Figure 4A and data not shown).

However, combination of the I12b enhancer deletion with that of I56i (Figure 4B) or with the I56i variant (Figure 4C), resulted in an additive or synergistic effects of the two mutations, with a more prominent phenotype at E13.5. The expression levels of *Dlx1*, *Dlx2*, *Dlx5*, and *Dlx6* were reduced by more than twofold (Figures 4B,C).

### GABA^+^ Neurons Are Decreased in the Developing Forebrain of I56i^(–/–)^ and vI56i Mutants

**FIGURE 5:**
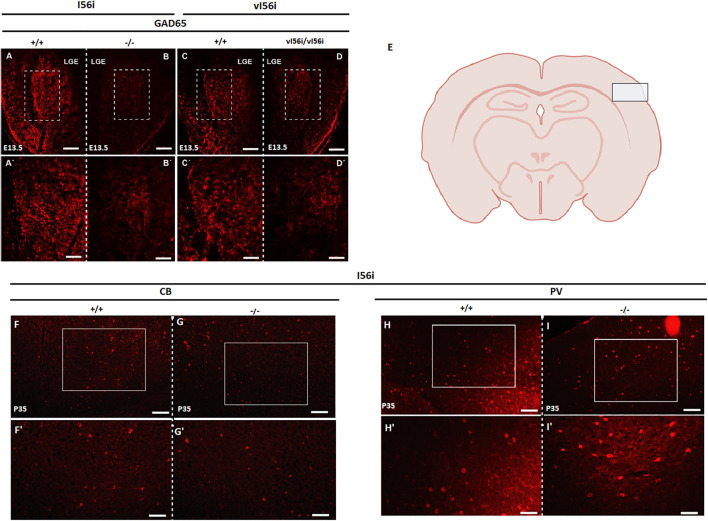
Mutations in the I56i enhancer decreases number of GABA^+^ cells at midgestation. **(A–D,A′–D′)** Immunostaining on E13.5 forebrain tissue with an anti GAD65 antibody. **(A,C)** WT, **(B)** homozygous I56i^(–/–)^, and **(D)** homozygous vI56i. Scale bar **(A–D)** = 100 μM and **(A′–D′)** = 50 μM. **(E)** schematic drawing of the mouse P35 forebrain, with a box indicating the position of the somatosensory cortex imaged. **(F,G,F′,G′)** Immunostaining for calbindin using an anti-calbindin antibody on P35 somatosensory cortex. **(F)** WT and **(G)** homozygous I56i^(–/–)^. **(H,I,H′,I′)** Immunostaining for parvalbumin using an anti-parvalbumin antibody on P35 somatosensory cortex. **(H)** WT and **(I)** homozygous I56i^(–/–)^. Scale bar **(F–I)** = 50 μm and **(F′–I′)** = 25 μm. LGE, lateral ganglionic eminence; CB, calbindin; PV, parvalbumin.

To examine whether intergenic enhancers contribute to *Dlx* role in neuronal differentiation, we performed immunohistochemistry for GABA, the main inhibitory neurotransmitter used by GABAergic interneurons in the developing forebrain of I56i^(–/–)^ and vI56i mutant mice (Figures 5A,B). We observed a noticeable decrease in GAD65 immunoreactivity in the lateral ganglionic eminence (LGE) of I56i^(–/–)^ and vI56i mice at E13.5 (Figures 5A–D). We then examined immunoreactivity for markers of various subtypes of GABAergic neurons in the somatosensory cortex of P35 mice (Figure 5E). However, no marked differences could be observed (Figures 5F–I). In fact, we could not observe differences in in the apparent number of GABAergic neurons at times later than E13.5 (data not shown) suggesting some compensatory mechanisms may be taking place. This is also consistent with the transient decrease in *Gad1* and *Gad2* expression (Figure 2 and data not shown) observed in ΔI56i mice.

### I56i^(–/–)^/ΔI12b^(–/–)^ Mutant Mice Exhibit Increased Sociability

Since the altered activity of the *Dlx* intergenic enhancers impact *Dlx* expression; we further examined whether this would cause behavioral deficits in the mutant mice. We examined mice that were homozygous for the I56i^(–/–)^ mutation, the vI56i mutation or that were homozygous for both one of the above mutation and the ΔI12b mutation. We did not observe any differences between homozygous mutant mice and their wild-type littermates in beam break [I56i: *t*-test, *t*(31.05) = −1.955, *p* = 0.06], elevated plus maze (I56i, time in open arm; one-way ANOVA, *p* = 0.77), and open field behavioral assays (I56i, time in large center, *t*-test; *p* = 0.057). Thus, there was a trend for higher activity and anxiety-like behavior in the I56i^(–/–)^ mice but this did not reach statistical significance.

**FIGURE 6:**
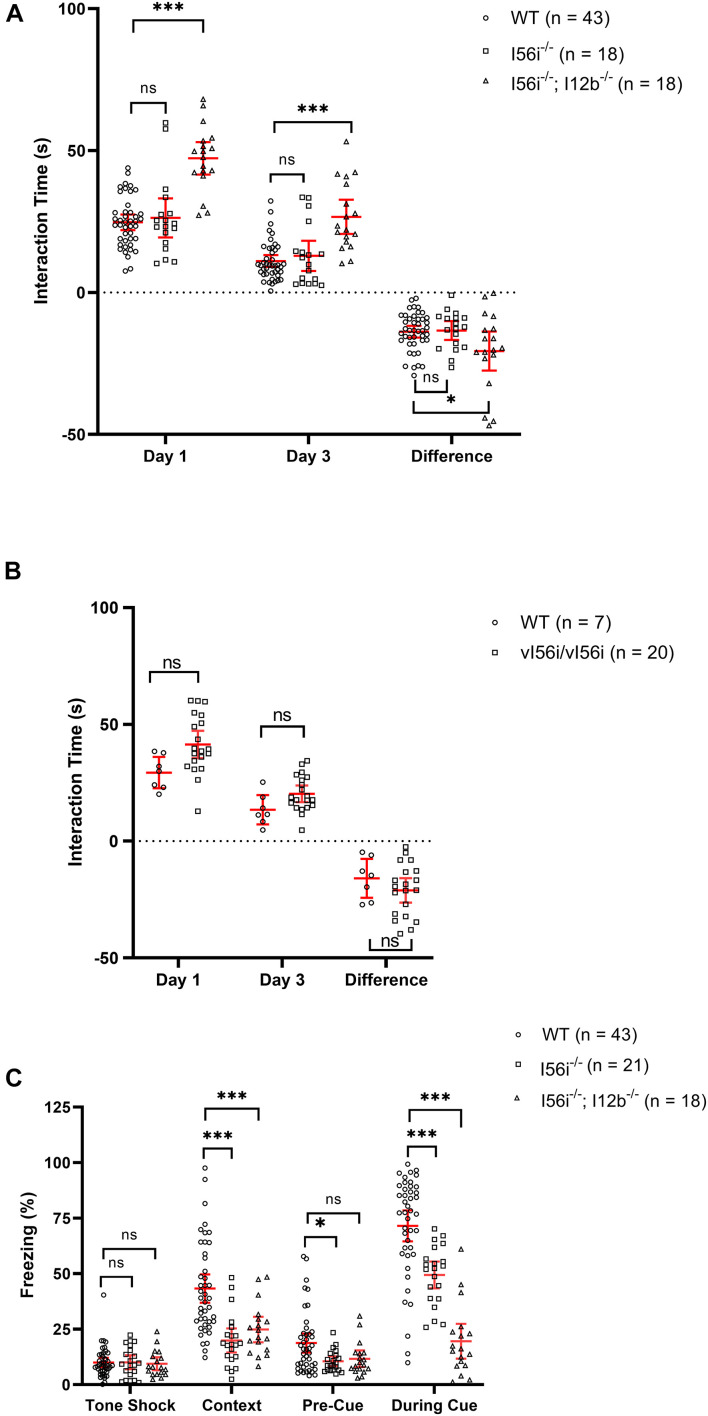
Mice with deletions of *Dlx* enhancers show an increased propensity for sociability and memory/learning defects. **(A)** Social interaction test performed on WT (black), homozygous I56i^(–/–)^ (white) and homozygous I56i^(–/–)^ – I12b^(–/–)^ (stripes). **(B)** Social interaction test preformed on WT (black) and homozygous vI56i (white). **(C)** Fear conditioning test preformed on WT (black) homozygous I56i^(–/–)^ (white) and homozygous I56i^(–/–)^ – I12b^(–/–)^ (stripes). Numbers of mice listed in the brackets of each graph. Data shown as mean, error bars represent SEM. Data was analyzed using one-way ANOVA with Turkey’s multiple comparison test (*p < 0.05, ***p < 0.001).

In a juvenile interaction test, mice homozygous for the I56i deletion showed a tendency to interact more with the juvenile although this difference did not reach statistical significance (Figure 6A). The time spent interacting with the juvenile decreased on day 3 of the test compared to day 1, for both the mutant and the wild-type littermates. However, when mice were homozygous for both the I56i and I12b deletions, there was a larger increase in time spend with the juvenile [Figure 6A, *p* = 10^–10^ on day 1 and 10^–7^ on day 3)], both on day 1 and on day 3 of the test. The difference between the 2 days was also slightly larger in the I56i^(–/–)^ + I12b^(–/–)^ double mutants.

Mice homozygous for the vI56i mutation showed results similar to those of I56i^(–/–)^ mutants with a clear trend for increased socialization that did not reach statistical significance (Figure 6B). We also performed a fear conditioning test on I56i^(–/–)^ mutant mice or on I56i^(–/–)^ + I12b^(–/–)^, homozygous mutants. Both I56i^(–/–)^ mice or I56i^(–/–)^ + I12b^(–/–)^ mice showed significant perturbations in learning/memory as their cued fear response was significantly reduced suggesting a lower hippocampal involvement (Figure 6C).

For all behavioral testing, potential sex-related differences were examined. We did not see differences between males and females for any of the genotypes. The only difference that was observed was a ∼15% higher activity in females compared to males in the beam break test (*p* < 0.05, *t*-test) when vI56 mice were tested but this higher activity was seen in females of both vI56 and wild-type littermates (data not shown).

## Discussion

We have deleted two highly conserved enhancers I56i and I12b, each located in the intergenic region of the *Dlx5/Dlx6* and the *Dlx1/Dlx2* bigene clusters, respectively. Loss of enhancer function, resulted in altered expression of the *Dlx* genes and of some of their targets, transient decreases in GABAergic neuron numbers and behavioral deficits. The phenotypes of the mice lacking enhancer function are milder than those of the mice lacking the *Dlx* genes themselves, perhaps due to the overlapping function of the CREs. Thus, mice lacking either I56i or I12b are viable and do not show any morphological abnormalities or size differences of the body or brain.

The I56i and I12b intergenic enhancers were chosen for this study as they were likely to show a major role in the regulation of *Dlx* expression in the developing forebrain. Both I56i and I12b show activity patterns in the SVZ and MZ of the lateral and medial ganglionic eminences (LGE and MGE) during forebrain development (Ghanem et al., 2007). The I56i and I12b enhancers are highly conserved throughout evolution and show very little sequence variation among vertebrate species (Zerucha et al., 2000; Ghanem et al., 2003). We previously proposed (Ghanem et al., 2007) that interneuron subtypes use distinct combinations of *Dlx* enhancers from the time they are specified through adulthood.

### Contributions of Additional *Dlx* Enhancers to Forebrain Development

The *Dlx5/Dlx6* intergenic region contains one additional sequence, I56ii, with enhancer activity. However, I56ii activity differs markedly from that of I56i or I12b. I56ii marks distinct a distinct population of neurons, known as corridor cells, that are located in the mantle of the LGE and MGE between E11.5 and E13.5 (Ghanem et al., 2008). However, unlike the other intergenic enhancers, I56ii does not label interneuron progenitors in the basal ganglia, nor tangentially migrating cells to the cortex at E13.5. Instead, I56ii-positive cells mark a subpopulation(s) of post-mitotic projection neurons that tangentially migrate from the LGE deep to the MZ of the MGE and reside between the SVZ and the globus pallidus during mid-gestation. Deletion of I56ii impairs expression of *Dlx* genes and that of potential targets, including *Gad2*, as well as striatal markers *Islet1*, *Meis2*, and *Ebf1* (Fazel Darbandi et al., 2016). In addition, I56ii deletion reduces both the binding of DLX2 in the *Dlx5/Dlx6* intergenic region and the presence of H3K9Ac at the *Dlx5/Dlx6* locus, consistent with the reduced expression of these genes (Fazel Darbandi et al., 2016).

Additional enhancers have been identified in the chromosomal region surrounding the mouse *Dlx5/Dlx6* bigene cluster (Brown et al., 2010; Birnbaum et al., 2012). However, none of these have shown activity in the forebrain. In addition to I12b, the *Dlx1/Dlx2* intergenic region contains the I12a enhancer, whose activity, in mice, is limited to the branchial arch region (Park et al., 2004). A forebrain enhancer, URE2, is located upstream of the *Dlx1* gene (Ghanem et al., 2007). Impact of its deletion on forebrain development, has yet to be determined.

### Impact of Enhancer Deletion on Gene Expression

In I56i^(–/–)^ mutant mice there is a drastic decrease (∼80%) of *Dlx5/Dlx6* expression in the developing forebrain (Figure 2). I56i contains DLX protein binding sites and we and others have suggested (Zerucha et al., 2000; Zhou et al., 2004; Poitras et al., 2007) that auto and/or cross-regulation mechanisms may be an important part of enhancer function. Consistent with these results, *Dlx1/Dlx2* mutant mice show a substantial decrease in *Dlx5* and *Dlx6* expression (Zerucha and Ekker, 2000) reinforcing the important role DLX proteins play in the maintenance of their own expression. We cannot rule out that impaired *Dlx* expression in enhancer mutants may be a result of the loss of binding sites for other, yet unidentified, proteins.

### Impact of Enhancer Deletion on GABAergic Neurons

Loss of the I56i enhancer (Figure 2) leads to reductions in the mRNA levels of *Gad1* and *Gad2*, coding for glutamic acid decarboxylase, the enzyme responsible for GABA synthesis. However, these decreases are transient. The number of GABA positive cells show an apparent decrease in the mutant mice when compared to the wild-type littermates at E13.5 (Figure 5). This could imply that these mice might have a delayed or disrupted GABAergic neuronal development. The I12b and I56i intergenic enhancers are specifically active in somatostatin-, vasoactive intestinal peptide-, and calbindin-positive interneurons (Ghanem et al., 2007). We were not able to detect any major losses of GABAergic interneuron populations at later stages, at least when tested with markers such as calbindin or parvalbumin. Although we cannot rule out that minor populations are affected, the degree of recovery from the large decreases seen at E13.5 indicate some form of compensatory mechanism. However, these transient losses could have long-lasting effects on neural circuits as behavioral phenotypes are observed in the enhancer deletion mutants.

### Mice With Mutations in I56i Enhancer Exhibit Increased Sociability

It has been suggested that any imbalance in GABAergic circuitry may result in an increased excitatory state, leading to neuropsychiatric diseases such as Rett syndrome, autism, and anxiety (Acosta and Pearl, 2003). Given the importance of the *Dlx* genes in regulating the migration and development of GABAergic interneurons in the developing forebrain, we performed behavioral test on the mutant mice and have shown that the absence of the I56i enhancer in mice is associated with increased sociability and impaired fear conditioning (Figure 6).

Mice lacking both the I56i- and the I12b enhancer show increased sociability in a juvenile interaction test. On both day 1 and day 3 of the test, mutant mice spend more time with the juvenile. The difference from day 1 to day 3, was only slightly larger in the double mutants compared to their wild-type littermates. A SNP in the I56i enhancer had been identified in a family with cases of autism. We previously showed that this SNP impacts activity of the I56i when tested in transgenic mice with reporter constructs (Poitras et al., 2010). Thus, the SNP produced a reduction in enhancer activity, predominantly, in the medial and caudal ganglionic eminences and in streams of neurons tangentially migrating to the cortex (Poitras et al., 2010). Here, we more directly tested the functional importance of this sequence variant by “knocking it in” the *Dlx5/Dlx6* locus. We saw that vI56i mice showed a trend to increased sociability in this juvenile interaction test.

This SNP in I56i may be rare and a link with autism has not been found in all association studies (Grove et al., 2019). The SNP falls into an ultraconserved sequence that is identical in all vertebrates examined thus far even in species such as human and zebrafish that are separated by more than 400 million years of evolution (Zerucha et al., 2000; Ghanem et al., 2003). Therefore, it was very surprising to find such a polymorphism within a human population.

Although autism is often associated with decreased social interest, the increased sociability in I56i^(–/–)^ mice can be related to its relationship with Gtf2i a factor whose gene is located on human chromosome 7 in a region that is deleted in cases if Williams–Beuren syndrome (Francke, 1999), a neurodevelopmental, autism spectrum disorder characterized by overfriendliness and an increased trust in strangers (Francke, 1999). Our biochemical analysis of I56i (Poitras et al., 2010) identified factors that bind to the region of I56i where the SNP was found. Interestingly, one of the main factors binding to this region is *Gtf2i*. Similarly a recent study identified GTF2I as one of the factors that may have contributed to the stereotypical sociability observed in domestic dogs (vonHoldt et al., 2017). Thus, our observation that mice with mutations in the I56i intergenic enhancer, which would presumably affect GTF2I binding, show greater interest in the novel mouse during a test of juvenile interactions provides further evidence for a role of GTF2I contribution to the regulation of the *Dlx5/Dlx6* locus in establishing proper social behavior.

Fear conditioning measures learning and memory through association with predicting aversive events. In mammals, fear conditioning related to learning is a highly complex system regulated in a part by the amygdala and hippocampal complex (Phelps, 2004). The hippocampus plays a primary role and is essential in episodic memory, which is the primary memory system in mammals that allows recollection of certain events at individuals will. Examination of the impact of *Dlx* enhancer deletion on interneuron populations in the hippocampus deserves further investigation. Amygdala function is involved in more long-term storage of associated emotional events (Phelps, 2004). The amygdala is involved in fear conditioning responses to simple modality-specific conditioned stimuli and plays an associative role, while the hippocampus is solely involved in memory of more complex polymodal events and plays more of a sensory relay role (Phillips and LeDoux, 1992). Similar to the I56i mutants, mice lacking *Dlx1* function also have reduced fear conditioning inhibitions (Mao et al., 2009). Overall, these results suggest that the absence of the I56i contributes to transient GABAergic dysfunction which may have implications in neurological disorders.

Studying highly conserved regulatory elements involved in the *Dlx* regulatory network will not only contribute to an enhanced knowledge of the pathways involved in regulating *Dlx* expression but will shed light on the underlying mechanisms involved in neurological disorders associated with disrupted GABA circuitry.

## Data Availability Statement

The raw data supporting the conclusions of this article will be made available by the authors, without undue reservation.

## Ethics Statement

The animal study was reviewed and approved by University of Ottawa Animal Care Committee.

## Author Contributions

LP and ME: conceptualization. SF, CE, CL-P, SM, LP, SP, MY, GH, and ME: formal analysis. SF, CE, CL-P, SM, LP, MY, and GH: methodology. LP, SP, and ME: supervision. SF, SM, and ME: validation and writing – review and editing. SF and ME: writing – original draft. All authors have read and agreed to the published version of the manuscript.

## Conflict of Interest

The authors declare that the research was conducted in the absence of any commercial or financial relationships that could be construed as a potential conflict of interest.

## Publisher’s Note

All claims expressed in this article are solely those of the authors and do not necessarily represent those of their affiliated organizations, or those of the publisher, the editors and the reviewers. Any product that may be evaluated in this article, or claim that may be made by its manufacturer, is not guaranteed or endorsed by the publisher.
